# Effect of tamoxifen in patients with thin endometrium who underwent frozen–thawed embryo transfer cycles: a retrospective study

**DOI:** 10.3389/fendo.2023.1195181

**Published:** 2023-09-01

**Authors:** Mengxia Ji, Xiaohua Fu, Danni Huang, Ruifang Wu, Yunqing Jiang, Qiongxiao Huang

**Affiliations:** ^1^ Center for Reproductive Medicine, Department of Reproductive Endocrinology, Zhejiang Provincial People’s Hospital, Hangzhou Medical College, Hangzhou, Zhejiang, China; ^2^ Department of Gynaecology, Tongxiang First People’s Hospital, Jiaxing, Zhejiang, China

**Keywords:** tamoxifen, thin endometrium (TE), frozen embryo transfer (FET), hormone replacement therapy (HRT), *in vitro* fertilization

## Abstract

**Introduction:**

Thin endometrium leads to an impaired implantation rate. The aim of the study is to compare the clinical outcomes of tamoxifen (TAM) and hormone replacement therapy (HRT) used in patients with thin endometrium (<7mm) in frozen-thawed embryo transfer (FET)cycles.

**Methods:**

A total of 176 FET cycles with thin endometrium were retrospectively analyzed in our center from Jan 2020 to May 2022. According to patients' own will, 112 patients were allocated to the HRT group and 64 patients chose the TAM protocol. Clinical outcomes were compared between two groups.

**Result:**

The duration of treatment was shorter in the TAM group(12.03±2.34d) than the HRT group (16.07±2.52 d), which was statistically different (p<0.05). The endometrial thickness on the transfer day of the TAM group (7.32±1.28 mm) was significantly thicker than that of the HRT group (6.85±0.89mm, p<0.05). The clinical pregnancy rate of the TAM group (50.0%) was higher than that of the HRT group (36.6%), but there was no significant difference (p >0.05). The early miscarriage rate was significantly lower in the TAM group compared with the HRT group (5.9% Vs 26.8%, adjusted OR 0.10, p<0.05), while the live birth rate was higher in the TAM group (46.9% Vs 26.8%, adjusted OR 2.24, p<0.05) compared with the HRT group.

**Conclusion:**

For patients with thin endometrium, TAM effectively improved the endometrial thickness and increased the live birth rate. TAM can be used as an alternative protocol for patients with thin endometrium.

## Introduction

Good endometrium receptivity plays a vital part in embryo implantation. It is well-acknowledged that endometrial thickness is associated with clinical outcomes in embryo implantation (1–3). Though the thicker, the better is not applicable in this situation, the thickness of endometrium below a certain limit will definitely affect the implantation rate (4) (5). In addition, a thin endometrium also gives rise to obstetric complications and adverse perinatal outcomes (6).

How thin is thin? The most acceptable cutoff value for thin endometrium is 7 mm on ultrasound (7–11). According to statistics, the prevalence of thin endometrium is varied from 2.4% (8) to 5.5% (12). Various factors may lead to thin endometrium, including iatrogenic injury, inflammation, and drug-induced and idiopathic factors (5). Damage of the basal layer of endometrium, poor vascularity, and low level of estrogen can all lead to suboptimal endometrial growth (4, 13). Thus, corresponding to pathophysiological mechanisms mentioned above, solutions from different perspectives were put forward to stimulate endometrium growth (14). One of the most frequently used methods is to increase the doses of estrogen or extend the duration of estrogen administration (15). The second choice is to improve uterine blood flow by various medications including aspirin, sildenafil citrate, and pentoxifylline-tocopherol (13). Infusion of growth factors into the uterine such as G-CSF (16) or platelet-rich plasma (PRP) (17) has been an innovative initiative proposed in recent years. Endometrial cell reconstruction by stem cells may also be a promising direction (18). Nevertheless, the results of these studies are controversial and none of these strategies are proved to be “a final solution” to thin endometrium.

As a kind of nonsteroid selective estrogen receptor (ER) modulators, tamoxifen (TAM) is an important anti-hormonal treatment for breast cancer patients with positive ERs (19, 20). However, long-term use of TAM will lead to an elevated risk of endometrial lesions, such as hyperplasia, polyps, and sometimes even carcinoma, which all originated from its proliferative effect on the endometrium (20, 21). Actually, TAM plays opposite roles in different organs. In the breast cancer, it can suppress tumor growth by antiestrogen action, while in the endometrium, its estrogen agonist effect is dominant and thus would stimulate endometrium proliferation. Moreover, TAM and clomiphene citrate (CC), the classical ovulation induction medicine, both belong to the family of triphenylethylene compounds (22). A number of studies showed that TAM was also effective in ovulation induction (22–26). The British Medical Association and Royal Pharmaceutical Society had licensed TAM use for infertility treatment in the United Kingdom (27). In a prospective study, the scholars found that TAM was as effective as CC in ovulation induction, and yielded a better endometrial thickness (28). Another study carried out by Reynolds et al. suggested TAM could be used in subsequent cycles among patients who have adequate follicular recruitment but thin endometrium (<7 mm) with CC to improve the endometrium thickness (25).

In recent years, TAM has been applied in endometrial preparation among patients with thin endometrium, and encouraging results were achieved (29–31). In this retrospective study, we compared the clinical outcomes of TAM with hormone replacement therapy (HRT) for patients with thin endometrium.

## Methods and materials

### Patients

From January 2020 to May 2022, a total of 176 FET cycles with a history of thin endometrium were included in this study. The inclusion criteria were as follows: age ≤40 years; the thickness of endometrium was <7 mm on the trigger day (fresh cycles) or the ovulation day (natural cycles) for at least two cycles; at least one embryo of top quality was transferred; endometrium preparation protocols should be TAM or HRT; no adjuvant use of aspirin, sildenafil citrate and pentoxifylline-tocopherol, G-CSF, and PRP. Female patients with abnormal karyotypes, uterine malformation, and a history of tuberculous endometritis were all excluded from this study. Before treatment, all female patients were fully informed of the novel use of TAM, including the indication, regimen, mechanism, and the possible birth defects concerning exposure to TAM before pregnancy. TAM was only used in those patients who wished to try this novel protocol and signed the informed consents. The study was approved by the ethics committees of Zhejiang Provincial People’s Hospital (grant number: 2020KT006).

### HRT cycles

For HRT cycles, estradiol valerate (Delpharm Lille S.A.S) was administered orally at a dose of 6 mg daily starting from the third day of menstrual cycle. After 7 days, vaginal estradiol (Femoston, Solvay pharmaceuticals B.V.) 0.5–1 mg/day were added. After 12–20 days of treatment, if the thickness of endometrium was unchanged for 2 consecutive days, progesterone (Zhejiang Xianju Pharmaceutical Co. Ltd) was injected at a dose of 40 mg per day.

### TAM cycles

For TAM cycles, TAM (Yangtze River Medicine, China) was given at a dose of 20–40 mg on the third day of the menstrual cycle for 5 days. Follicle monitoring was initiated on the 10th day of the menstrual cycle until the dominant follicle was ovulated. Serum hormone, including luteinizing hormone (LH), estradiol hormone (E_2_), and progesterone (P), was tested when the diameter of follicle ≥14 mm. If no dominant follicles appeared until the 20th day of the period, the cycle was cancelled. Progesterone was started on the ovulation day at the same dose as the HRT protocol.

### Endometrium measurement and FET procedures

To minimize the difference of inter-observers, every endometrial measurement was performed by two experienced observers according to the same criteria. The thickest echogenic area from one stratum basalis endometrial interface across the endometrial canal to the other stratum basalis interface in the sagittal plane was defined as the endometrial thickness (5). All measurements were repeated at least 2 times, and the mean value of two observers was recorded for analysis. The progesterone priming day was defined as Day 0. Cleavage stage embryos were transferred on Day 3 and blastocyst transfer was performed on Day 5. The period from estrogen/TAM administration to progesterone add-up was defined as the endometrium preparation duration.

### Embryo grading

The cleavage stage embryos were morphologically assessed including the number, size, distribution of blastomeres, and cytoplasmic fragmentation percentage (32). Embryos with at least 7cells and graded 1 and 2 on day 3 were defined as top-quality. The blastocyst evaluation was adopted using the Gardner grading system (33). Blastocysts graded higher than 3BB on day 5 or 4BB on day 6 were deemed as top-quality blastocysts.

### Pregnancy outcome assessment

Blood HCG level was tested 12 days after FET, and transvaginal ultrasound was performed 35 days after FET. If a gestational sac was detected, clinical pregnancy was confirmed. Spontaneous miscarriage in the first trimester was deemed as early miscarriage. Viable neonates delivered after 28 weeks of gestation were defined as live births. The gestational age, the neonates’ birth weight, height, and preterm birth were also followed up.

The implantation rate was defined as the number of embryos implanted divided by the number of embryos transferred. The calculation of the clinical pregnancy rate, the early miscarriage rate, and the live birth rate were all based on the total number of FET cycles.

### Statistical analysis

Statistical analysis was performed by SPSS (Chicago, IL, USA version 21). All measurement data were expressed as mean ± standard deviation. For continuous variables with normal distribution, Student’s *t* test was adopted. Pearson Chi-square test was applied to proportion comparisons among groups. To adjust for confounders, a binary logistic regression analysis was performed and the results were reported as adjusted odds ratios (aORs) with 95% CI. A value of *p* less than 0.05 was defined as statistically significant.

## Results

### Baseline data

Of the 176 cycles, 112 patients underwent HRT cycles and 64 patients adopted TAM (**Table 1**). The baseline data, including the average female age, AMH, BMI, duration of infertility, and causes of infertility were comparable between the two groups (*p* > 0.05); 73.2% of the patients had undergone uterine operation (including dilation and curettage, and hysteroscopy) in the HRT group, while the percentage in the TAM group was 81.3% (*p* > 0.05). The proportion of patients with a history of intrauterine adhesion in the HRT group and TAM group were 28.6% and 37.5%, respectively (*p* > 0.05). The previous endometrial thickness was significantly higher in the HRT group (6.58 ± 0.45 mm) than in the TAM group (6.33 ± 0.67 mm, *p* = 0.01). Moreover, the frequencies of previous implantation failures and pregnancy loss were also similar in both groups (**Table 1**).

**Table 1:** Baseline demographics and cycle characteristics between the two groups.

Variables	HRT (*n* = 112)	TAM (*n* = 64)	*p*
Age (years)	32.03 ± 3.57	31.79 ± 4.18	0.69
Infertility duration (years)	3.09 ± 2.37	2.60 ± 2.00	0.17
AMH (μg/ml)	4.09 ± 3.53	3.68 ± 3.18	0.44
BMI (kg/m^2^)	21.82.21 ± 3.02	21.45 ± 2.37	0.40
Causes of infertility (%)			
Tubal factor	51.8 (58/112)	56.3 (36/64)	0.49
Male factor	19.6 (22/112)	26.6 (17/64)	
Ovulation disorder	17.0 (19/112)	10.9 (7/64)	
Endometriosis	4.5 (5/112)	1.6 (1/64)	
Unknown	7.1 (8/112)	4.7 (3/64)	
Previous endometrial thickness (mm)	6.58 ± 0.45	6.33 ± 0.67	0.01
Proportion of patients with a history of uterine operation* (%, *n*/N)	73.2 (82/112)	81.3 (52/64)	0.27
Proportion of patients with intrauterine adhesion (%, *n/N*)	28.6 (32/112)	37.5 (24/64)	0.22
Proportion of patients with a history of pregnancy loss (%, *n*/*N*)	22.2 (25/112)	18.8 (12/64)	0.57
Frequencies of previous implantation failures (%, *n*/*N*)			0.75
0-2	93.8 (105/112)	95.3 (61/64)	
≥3	6.3 (7/112)	4.7 (3/64)	

*Including dilation and curettage, and hysteroscopy.

During the treatment, the TAM group had fewer days of endometrium preparation (12.03 ± 2.34 days) than the HRT group (16.07 ± 2.52 days, *p* = 0.00). Though the concentration of E_2_ on the transfer day was significantly higher in the HRT group (500.26 ± 294.77 pg/ml) than the TAM group (230.33 ± 157.80 pg/ml, *p* = 0.00), the endometrial thickness of the HRT group (6.85 ± 0.89 mm) was significantly lower than that of the TAM group (7.32 ± 1.28 mm, *p* = 0.00). Meanwhile, patients in the TAM group showed significantly higher concentration of P than those in the HRT group (26.09 ± 8.88 ng/ml vs. 11.64 ± 6.72 ng/ml, *p* = 0.00). The average number of embryos transferred was 1.41 ± 0.49 and 1.48 ± 0.50 in the HRT group and the TAM group, respectively (*p* > 0.05). The proportion of patients who underwent single embryo transfer and cleavage embryo transfer were comparable in both groups (**Table 2**).

**Table 2:** The comparison of clinical outcomes between the two groups.

Variables	HRT (*n* = 112)	TAM (*n* = 64)	*p*
Duration of endometrium preparation (days)	16.07 ± 2.52	12.03 ± 2.34	0.00
Embryo transfer day			
E_2_ (pg/ml)	500.26 ± 294.77	230.33 ± 157.80	0.00
P (ng/ml)	11.64 ± 6.72	26.09 ± 8.88	0.00
Endometrial thickness (mm)	6.85 ± 0.89	7.32 ± 1.28	0.01
No. of embryos transferred (%)			0.50
Single	58.9 (66/112)	51.6 (33/64)	0.52
Double	41.1 (46/112)	48.4 (31/64)	
Embryo stage at transfer			
Cleavage stage	64.3 (72/112)	59.4 (38/64)	0.74
Blastocyst stage	35.7 (40/112)	40.7 (26/64)	
No. of embryos per transfer	1.41 ± 0.49	1.48 ± 0.50	0.35
No. of top-quality embryos per transfer	1.17 ± 0.63	1.22 ± 0.70	0.63
Implantation rate (%)	29.7 (47/158)	38.9 (37/95)	0.22
Clinical pregnancy rate (%)	36.6 (41/112)	50.0 (32/64)	0.11
Early miscarriage rate (%)	26.8 (11/41)	5.9 (2/32)	0.02
Live birth rate (%)	26.8 (30/112)	46.9 (30/64)	0.008

Concerning the clinical outcomes (**Tables 2**, **3**), we found that both the implantation rate (38.9% vs. 29.7%) and the clinical pregnancy rate (50.0% vs. 36.6%) were higher in the TAM group than in the HRT group, but no statistical difference was found (*p* > 0.05). The early miscarriage rate was significantly lower in the TAM group than in the HRT group (5.9% vs. 26.8%, aOR = 0.10, *p* = 0.03). Moreover, the live birth rate of the TAM group (46.9%) was significantly higher than that of the HRT group (26.8%, aOR = 2.24, *p* = 0.04).

**Table 3:** Crude and adjusted odds ratios (ORs) of pregnancy outcomes between two groups.

Variables	Crude OR (95% CI)	*p*	Adjusted OR (95% CI)	*p*
Clinical pregnancy rate	1.73 (0.93–3.23)	0.08	1.445 (0.7–2.96)	0.32
Early miscarriage rate	0.19 (0.04–0.91)	0.03	0.10 (0.01–0.77)	0.03
Live birth rate	2.41 (1.27–4.60)	0.007	2.24 (1.05–4.75)	0.04

Binary logistic regression analysis.

ORs adjusted for maternal age, BMI, duration of infertility, infertility diagnosis, the number and stage of embryos transferred, and the endometrial thickness, with the HRT group as the reference.

**Table 4** shows the neonatal outcomes of the two groups. The average gestational age, birth weight, and birth height were comparable in the HRT group and the TAM group. Six newborns of the HRT group (20%) were premature, while in the TAM group, the preterm birth was 4 (13.3%, *p* > 0.05). None of the babies were born with birth defects in both groups.

**Table 4:** The follow-up of neonates in the two groups.

Variables	HRT group (*n* = 30)	TAM group (*n* = 30)	*p*
Gestational age (days)	263.2 ± 20.02	265.33 ± 14.59	0.64
Birth weight (g)	2736.18 ± 815.65	2836.71 ± 663.34	0.31
Birth height (cm)	48.18 ± 7.53	48.05 ± 2.52	0.29
Preterm birth (%, *n*/N)	20.0 (6/30)	13.3 (4/30)	0.49
Birth defect	0	0	/

## Discussion

At first, TAM was used in ovulation induction in women who underwent intrauterine insemination (IUI) whose endometrial thickness was less than 8 mm in previous ovulatory cycles (22). Using a dose of 40 mg of TAM on the 3rd day of the period for 5 days, the author found that not only the endometrial thickness was greatly improved (6.7 ± 1.3 mm and 10.8 ± 2.3 mm, *p* < 0.001), but also the clinical pregnancy rate was significantly elevated (32.1% vs. 15.9%, *p* = 0.014) compared with the group stimulated by CC.

Encouraged by this report, Tian et al. applied TAM in female patients who underwent FET with thin endometrium (30) (published in Chinese, see **Supplementary Material 1**). They included 61 patients who failed to develop adequate endometrial thickness (<7 mm) for at least two previous FET cycles. TAM was stared on the 3rd to 5th day of the menstruation for 5 days. Ultimately, ovulation occurred in 83.6% of the patients (51/61). Though dominant follicles did not develop in 10 cases, FET was still performed when the endometrial thickness exceeded 8 mm. In total, the thickness of endometrium was improved in 95.1% of cases, and 44.3% (27/61) of patients got pregnant after this novel protocol. However, this study was a retrospective observational study without control groups. Whether TAM had an advantage over classical HRT or natural cycles still needed to be explored.

In 2016, Liu et al. compared the effect of TAM and HRT in patients with thin endometrium (<8 mm) (31) (published in Chinese, see **Supplementary Material 2**). The endometrial thickness of the TAM group on the transfer day was significantly higher than that of the HRT group (8.2 ± 1.1 mm vs. 7.1 ± 0.3 mm, *p* < 0.05). The implantation rate (34.6% vs. 28.7%) and clinical pregnancy rate (48% vs. 38.5%) were also improved in the TAM group in cleavage embryos transfer while no difference was observed in blastocyst transfer. The author attributed the inconsistency between different stages of embryo transfer to the better potential of blastocysts and the small sample size of the blastocyst transfer group (*n* = 78).

Another report concerning TAM application in FET among patients with thin endometrium (<8 mm) was published by Ke et al. in 2018 (29). They divided the patients into three groups according to different causes of thin endometrium, the intrauterine adhesion (IUA) group, the uterine curettage (UC) group, and the polycystic ovary syndrome (PCOS) group. The endometrial thickness was improved in all three groups. The clinical pregnancy rate and live birth rate was highest in the PCOS group (60%, 55.56%), followed by the UC group (38.61%, 31.68%) and the IUA group (33.33%, 27.78%), indicating that TAM may perform better in patients whose endometrium was not injured. Regretfully, in this study, 17 β estradiol or estradiol valerate was concomitantly used in the TAM protocol; thus, the improved outcomes could not be attributed to TAM alone.

The three aforementioned studies (29–31) were literature from which we can obtain the effect of TAM in endometrium preparation in FET so far, and two of them were published only in Chinese (30, 31). The results of our study were consistent with those of previous studies in that a better thickness of endometrium was obtained after TAM (7.32 ± 1.28 mm vs. 6.85 ± 0.89 mm, *p* < 0.05). Though the previous endometrial thickness was thinner with a lower level of E_2_ in the TAM group, it grew significantly thicker than the HRT group on the transfer day, suggesting that TAM was advantageous in stimulating endometrium growth for patients with thin endometrium. The underlying mechanisms are complicated. TAM not only upregulated the expression of ER, but also promoted the expression of cell proliferation markers such as Ki67 and IGF-1 (34–36). Moreover, TAM could induce the local estrogen biosynthesis through altering estrogen-metabolizing enzymes (37). Resistance (RI) and pulsatility (PI) indices of the uterine arteries that decreased after TAM treatment might also contribute to the growth of endometrium (38). Therefore, Tian et al. (30) observed that the endometrium continued to grow thicker upon TAM stimulation without a leading follicle appearing. Nevertheless, in our study, the anovulation cycles of TAM were canceled. Meanwhile, the significantly shortened duration of endometrium preparation of TAM made it more friendly to patients.

Regarding the clinical outcomes, we found that the implantation rate and clinical pregnancy rate of were comparable in the two groups. Nevertheless, compared with the HRT group, the early miscarriage rate of the TAM group was significantly lower (5.9% vs. 26.8%, aOR = 0.10, *p* = 0.03) and the live birth rate (46.9% vs. 26.8%, aOR = 2.24, *p* = 0.04) was significantly higher than that of the HRT group before and after adjusting for confounders. In previous studies concerning the effects of TAM and CC in ovulation induction, it was found that TAM was associated with a lower risk of miscarriage rate (22, 39). An increased vessel density and a relatively better vascularity induced by TAM might partly explain the better outcome of TAM (40). In addition, the corpus luteum formed after ovulation induced by TAM could secrete higher concentration of progesterone than the HRT protocol. Recently, it was reported that besides E_2_ and P, corpus luteum may also secrete vasoactive products such as relaxin and vascular endothelial growth factor (VEGF) (41), which were conducive to embryo implantation. Yet, this result should be interpreted cautiously since this study was retrospective with a small sample size. Additionally, embryos became a confounding factor in this study though the average number and the proportion of developmental stages of embryos were comparable between groups. Consequently, prospective trials with large samples are still needed to further explore the value of TAM in patients with thin endometrium.

The primary concern of TAM use is its safety in women preparing for pregnancy. According to statistics, 13 infants were born with congenital malformations of the 142 live births documented (42). When pregnancies with documented fetal outcomes were all included, the incidence of fetal defects was 12.6% (12/167) (42), which was significantly higher than the general population (3.9%) (41). Nevertheless, no certain kind of malformation was related to TAM, and the concomitant medications were not all documented (43). In addition, all the malformation reports were from patients with breast cancer, who were required to take TAM as a long-term therapy since the serum concentrations of TAM were steady after 4 weeks of use (44). Thus, the elimination half-life was as long as 7 days, and some scholars recommended 2 months of washout period after TAM withdrawal (27, 43). Unlike the long-term use in breast cancer, TAM was administered only for 5 days and stopped at least for 7 days before embryo transfer in our study. Frozen embryos developed at previous cycles, which was irrelevant to TAM. In our follow-up, we found that the average gestational age, birth weight, and birth height were all comparable in the two groups. More importantly, no birth defect of neonates was seen from patients in the TAM group. Close follow-up was still needed in the future.

The retrospective design was the main limitation of our study. Since the groups were not divided by randomization, there may be an uneven distribution of cases between groups, which would lead to a weakened comparability of baseline variables. The embryos transferred were not controlled at the same level, which would also undercut the strength of the result.

## Conclusion

In our study, for women with thin endometrium, we found that TAM effectively improved the endometrial thickness. The implantation rate and clinical pregnancy rate were similar in the two groups. The early miscarriage rate was significantly reduced and the live birth rate was elevated after TAM treatment. TAM is more than just a type of breast cancer lifesaving drug; it may also be a blessing for those who underwent FET with thin endometrium.

## Data availability statement

The raw data supporting the conclusions of this article will be made available by the authors, without undue reservation.

## Ethics statement

The studies involving humans were approved by the ethics committees of Zhejiang Provincial People’s Hospital(grant number:2020KT006). The studies were conducted in accordance with the local legislation and institutional requirements. The participants provided their written informed consent to participate in this study.

## Author contributions

All authors contributed to the study. Study conception and design, data collection, analysis, and manuscript writing were performed by MJ. XF contributed significantly to data analysis; DH helped to collect and process the data. RW and YJ contributed to clinical practice. QH supervised the whole study and helped with manuscript revision. MJ obtained fund and supported this research. All authors have read and approved the final manuscript.
