# Supplementary material for: Effect of tamoxifen in patients with thin endometrium who underwent frozen–thawed embryo transfer cycles: a retrospective study
Source: Front Endocrinol (Lausanne). 2023 Sep 1;14:1195181. doi: 10.3389/fendo.2023.1195181 (PMC10505727; doi:10.3389/fendo.2023.1195181)
Supplement: Supplementary file 1 [file DataSheet_1.pdf]

论　著

文章编号:1005-2216(2015)08-0736-05

他莫昔芬用于薄型子宫内膜患者冻融胚胎移植时子宫内膜准备的研究

田小龙,陈　薪,许丽娟,叶德盛,刘玉东,王　楠,郭萍萍,陈士岭

摘要:目的　探讨他莫昔芬(tamoxifen)方案应用于以往自然周期和激素替代治疗反复出现子宫内膜薄的患者行冻融胚胎移植(FET)时子宫内膜准备的临床效果。**方法**　2011年11月至2014年4月南方医科大学南方医院妇产科生殖医学中心,回顾性分析61例既往因自然周期和激素替代治疗准备冷冻周期子宫内膜,显示子宫内膜薄而取消移植或者移植后未妊娠患者使用他莫昔芬治疗,观察子宫内膜厚度、性激素水平以及FET后的妊娠结局。**结果**　他莫昔芬周期和既往激素替代治疗周期相比,前者的子宫内膜厚度[(8.8±1.8)mm、(6.5±0.3) mm]和血清雌二醇(E_2)水平[(2091.2±1020.5)pmol/L、(969.6±474.2)pmol/L]明显较高,且差异具有统计学意义($P<0.05$);子宫内膜准备天数明显减少[(13.2±2.9)d、(23.1±4.9)d],差异有统计学意义($P<0.05$)。他莫昔芬周期的临床妊娠率、早期流产率、胚胎种植率、畸形率分别为44.3%(27/61)、7.4%(2/27)、24.2%(32/132)、0(0/17)。**结论**　对于子宫内膜薄的患者,在冻融胚胎移植周期使用他莫昔芬准备子宫内膜方法简单可行,而且子宫内膜准备时间较短、子宫内膜厚度显著提高,并可获得较满意的妊娠结局。

关键词: 他莫昔芬; 薄型子宫内膜; 冻融胚胎移植; 子宫内膜准备
中图分类号: R321-33　**文献标志码:** A

Effect of tamoxifen on clinical outcome of patients with thin endometrium undergoing frozen-thawed embryo transfer.

TIAN Xiao-long, CHEN Xin, XU Li-juan, YE De-sheng, LIU Yu-dong, WANG Nan, GUO Ping-ping, CHEN Shi-ling.　Center for Reproductive Medicine,Department of Gynecology and Obstetrics, Nanfang Hospital, Southern Medical University, Guangzhou 510515, China
Corresponding author: CHEN Shi-ling,E-mail: chensl_92@163.com

Abstract: Objective　To explore the effect of tamoxifen(TAM) on endometrium preparation in patients with thin endometrium for frozen-thawed embryo transfer (FET).**Methods**　Retrospectively analyze 61 patients visiting the Center for Reproductive Medicine, Department of Gynecology and Obstetrics,Nanfang Hospital, affiliated to Southern Medical University for IVF/ICSI from November 2011 to April 2014, who canceled embryo transfer (ET) or failed to have conception after FET due to thin endometrium in natural cycle or hormone replacement therapy cycle. In these patients TAM was used to prepare endometrium for FET. The endometrial thickness, levels of hormone, and clinical outcomes were analyzed.**Results**　The endometrial thickness and serum E_2 level in TAM cycles were significantly higher compared with those in hormone replacement therapy cycle [(8.8±1.8)mm vs. (6.5±0.3) mm,$P<0.05$;(2091.2±1020.5) pmol/L vs. (969.6±474.2)pmol/L,$P<0.05$]. Days to prepare the endometrium was shorter (13.2 ± 2.9 days vs. 23.1 ± 4.9 days), and the difference was statistically significant. The clinical pregnancy rate, early miscarriage rate, implantation rate, and deformity rate in TAM cycle were 44.3% (27/61), 7.4% (2/27), 24.2% (32/132), and 0% (0/17), respectively. **Conclusion**　It´s simple and feasible to use tamoxifen in patients with thin endometrium while undergoing frozen-thawed embryo transfer,which has short duration of endometrial preparation, significantly improves the endometrial thickness, and results in satisfactory pregnancy rate.

Keywords: tamoxifen;thin endometrium;frozen-thawed embryo transfer;endometrial prepration

冻融胚胎移植已经成为辅助生殖助孕的重要技术之一。它具有避开取卵周期中的子宫内膜异常、避免卵巢过度刺激综合征的发生、减少患者治疗费用、增加累计妊娠率及活产率等优点[1]。理想的子宫内膜厚度是确保胚胎种植成功的关键,一

DOI:10.7504/fk2015070114
基金项目:广州市科技计划项目重点项目(11C22120737);国家自然科学基金(81170574);南方医科大学南方医院高层次匹配课题基金(G201206)
作者单位:南方医科大学南方医院妇产科生殖医学中心,广东 广州 510515
通讯作者:陈士岭,电子信箱:chensl_92@163.com

般认为子宫内膜厚度在7 mm以下时,胚胎移植后妊娠率显著下降[2]。但是少部分患者采用常规子宫内膜准备方案却难以达到足够的厚度。为此,临床医生采用了各种方法试图提高患者子宫内膜厚度,包括增加雌激素使用剂量和延长雌激素使用时间;给予低剂量的阿司匹林、枸橼酸西地那非、神经肌肉电刺激等改善子宫血流;宫腔注射干细胞以及细胞因子等[3-4]。可是仍有部分患者在采用上述方法后子宫内膜的厚度未见提高。他莫昔芬(tamoxifen, TAM)是一种人工合成的非甾体类雌激素受体调节剂。作为治疗和预防乳腺癌的一线药物,TAM应用于临床已有几十年的历史。国内外文献报道中TAM多用于促排卵治疗或人工授精中可明显提高子宫内膜厚度[5],然而在冻融胚胎移植方面的应用却鲜有报道。本研究目的是对薄型子宫内膜不孕患者应用TAM进行冻融胚胎移植,探讨其在改善子宫内膜厚度及对临床妊娠结局的影响,为临床治疗薄型子宫内膜不孕患者提供参考。

1 资料与方法

1.1 研究对象 选择2011年11月至2014年4月间在南方医科大学南方医院妇产科生殖医学中心接受FET周期的患者61例。纳入标准:(1)年龄≤38岁。(2)移植胚胎数≥2个。(3)移植胚胎中至少有1个优质胚胎。(4)既往自然周期或者激素替代(hormone replacement therapy, HRT)周期准备子宫内膜反复出现(2次及以上)子宫内膜厚度<7 mm。排除标准:(1)供卵患者。(2)卵巢早衰累积胚胎移植患者。(3)在胚胎移植前加用其他促排卵药物或者雌激素患者。

1.2 子宫内膜准备方法

1.2.1 HRT周期子宫内膜准备方法 所有患者在前一周期均采用同一种外源性雌孕激素准备子宫内膜。患者于月经来潮第2天开始口服戊酸雌二醇(补佳乐,1 mg/片) 4 mg/d,每4天增加2 mg,共服12～14 d后超声监测子宫内膜厚度,子宫内膜厚度<7 mm者,补佳乐再增加至10 mg/d,最长使用时间不超过30 d,监测子宫内膜厚度和血E_2、P水平,若子宫内膜厚度仍<7 mm则取消移植。

1.2.2 TAM周期子宫内膜准备方法 患者于月经来潮第3～5天口服TAM片(10mg/片)20～40 mg,连续服用5 d,停药3 d起超声监测子宫内膜厚度和血清E_2、P水平,等待优势卵泡排卵后3 d或5 d移植D3或D5胚胎;若无优势卵泡生长,则在子宫内膜厚度>8 mm后抽血检测血清E_2、LH和P水平,再给予黄体酮类药物进行子宫内膜转化,于用药后3 d或5 d移植D3或D5胚胎。

1.3 黄体支持方案 黄体酮每日肌肉注射40 mg和地屈孕酮片每日口服2次,每次20 mg。

1.4 移植结局 生化妊娠:移植12 d血HCG≥25 U/L;临床妊娠:移植后4周超声下见到妊娠囊。妊娠≥13周为继续妊娠。种植率:超声下见到的妊娠囊总数占移植胚胎总数的百分比。畸形率:畸形胎儿占已分娩胎儿总数的百分比。

1.5 统计学分析 使用SPSS16.0软件进行统计分析,均数采用$\bar{x}\pm s$表示,计数资料采用Pearson χ^2检验,计量资料采用配对样本t检验,以$\alpha=0.05$为检验水准,$P<0.05$表示差异有统计学意义。

2 结果

2.1 患者的基本情况 患者年龄为30.5±2.8(24～38)岁,不孕年限4.4±2.3(1～10)年。其中原发性不孕患者有55.7%(34/61),继发性不孕患者有44.3%(27/61)。在主要导致不孕的因素中,女性盆腔输卵管因素所占比例最高为80.3%(49/61),男性因素8.2%(5/61),双方共同因素为4.9%(3/61),不明原因性不孕为6.6%(4/61)。既往移植胚胎次数1.2±1.2(0～4)次,既往移植胚胎2.5±0.4(0～11)个。

2.2 TAM与HRT周期子宫内膜厚度、子宫内膜准备时间以及激素水平的比较 TAM周期较HRT周期行子宫内膜准备时可获得更高子宫内膜厚度、较高激素水平、较短子宫内膜准备时间,差异均有统计学意义($P<0.000$)。见表1。

2.3 TAM周期的妊娠结局 TAM方案进行的61个周期冻融胚胎移植,平均移植胚胎(2.16±0.37)个。生化妊娠率为59.0%(36/61),临床妊娠率为44.3%(27/61),胚胎种植率为24.2%(32/132)。早期流产率为7.4%(2/27),异位妊娠(EP)2例,宫内外孕1例(宫内早期流产),异位妊娠率为11.1%(3/27),继续妊娠率为85.2%(23/27)。在已出生的17名婴儿中无一例出生缺陷。

2.4 TAM周期不同卵泡和子宫内膜厚度生长的妊娠结局 应用TAM后无论有无优势卵泡生长其妊娠率差异无统计学意义($\chi^2=0.690$,$P=0.738$)。应

用TAM后不同子宫内膜厚度的妊娠率之间差异无统计学意义（χ^2=2.344，P=0.310），子宫内膜厚度大于8 mm组其妊娠率高于其他两组。见表2。

表1 TAM周期与HRT周期治疗情况（黄体酮注射日）比较

类别	子宫内膜厚度(mm)	血清E_2水平（pmol/L）	血清P水平（nmol/L）	子宫内膜准备时间（d）
TAM周期	8.8±1.8	2091.2±1020.5	3.5±0.75	13.2±2.9
既往HRT周期	6.5±0.3	969.6±474.2	1.78±1.26	23.1±4.9
t值	11.0	3.4	2.5	−9.9
P值	＜0.000	＜0.000	0.08	＜0.000

表2 TAM周期子宫内膜厚度、卵泡生长与妊娠情况

类别	所占比例	妊娠率	P值
优势卵泡			
有	83.6%（51例）	43.1%（22/51）	0.872
无	16.4%（10例）	50.0%（5/10）	
子宫内膜厚度（mm）			
≥8	63.9%（39例）	51.3%（20/39）	
>7～<8	18.0%（11例）	27.3%（3/11）	0.343
≤7	18.0%（11例）	36.4%（4/11）	

2.5 用药后子宫内膜厚度的分布情况以及子宫内膜不同变化值的分布情况 应用TAM后58例（95.1%）患者的内膜厚度得到不同程度的改善，其中厚度增加值在1 mm以上有46例（75.4%），2 mm以上有32例（52.5%），最大值可达6 mm。

3 讨论

3.1 薄型子宫内膜定义和既往的处理方式 目前对薄型子宫内膜的定义尚不统一，一般认为子宫内膜厚度达到7～8 mm是胚胎种植的必要条件。有研究认为子宫内膜厚度小于8 mm时胚胎种植率和临床妊娠率显著降低[6]，我们的医疗团队之前研究表明当子宫内膜厚度小于7 mm时临床妊娠率降至23.1%，自然流产率显著上升。目前对于子宫内膜薄的不孕患者主要处理方式有三类：（1）增加雌激素用量、延长使用时间以及阴道用药以增加子宫局部药物浓度。（2）调节和增加子宫血运、减低循环阻力，如小剂量阿司匹林、西地那非、电刺激等。（3）刺激子宫内膜增生，如给予骨髓干细胞或白细胞如集落细胞刺激因子宫腔内注射。2008年的一篇综述认为以上方法似乎均不能有效提高子宫内膜的厚度[7]。

3.2 TAM既往在助孕治疗中的应用 TAM属于第一代选择性雌激素受体调节剂（selective estrogen receptor modulators，SERMs），其消除半衰期为86 h[8]，具有雌激素激动和拮抗雌激素的双重作用。这种作用取决于不同的种系、组织和基因表达类型，在子宫和骨组织则是上调雌激素受体的作用。辅助生殖治疗中，TAM主要用于PCOS的促排卵治疗或者行人工授精的不孕患者，而在冻融胚胎移植中的应用鲜有报道。2013年本中心首次报道了3例患者在促排卵周期、自然周期以及雌激素替代周期均因子宫内膜薄而取消胚胎移植，后经TAM准备子宫内膜，最终子宫内膜均得到改善获临床妊娠和分娩的案例[9]。虽然氯米芬（CC）和TAM的分子结构较为相似，都有雌激素拮抗作用，然而前者对子宫内膜的抗雌激素作用更明显，导致部分患者子宫内膜薄。Reynolds等[10]报道19例患者在氯米芬促排卵中，虽然有优势卵泡生长但是子宫内膜厚度仍然小于7 mm，后一周期改用TAM方案，所有患者的子宫内膜均得到有效改善［TAM组（8.8±1.3）mm，CC组（5.5±0.8）mm］（P<0.001）。随机对照试验也证明TAM用于促排卵治疗比CC更有利于子宫内膜的生长［（10.1±0.1）mm vs.（9.3±0.1）mm］[11]。近期有研究表明在来曲唑（letrozole）联合HMG行IUI时，添加TAM较未添加TAM的患者，使用HMG较少，子宫内膜厚度较厚，妊娠率提高。

3.3 TAM促进子宫内膜生长和胚胎着床的机理 我们的研究结果显示TAM对于自然周期或激素治疗周期出现子宫内膜薄的患者有良好的治疗

效果，推测与更高的雌二醇水平有关。不孕患者在TAM周期的子宫内膜厚度为(8.8±1.8)mm，显著大于既往HRT周期(6.5±0.3)mm，在TAM周期的血清E_2水平[(2091.2±1020.5)pmol/L]显著高于既往HRT周期(969.6±474.2)pmol/L，差异具有统计学意义(P<0.05)。TAM促进子宫内膜生长的分子机理是多方面的。有学者发现TAM可以促进体内雌激素整体水平的代谢以及在子宫内膜局部增加雌激素的合成[12]。TAM促进雌激素代谢可能与其刺激雌激素受体即G蛋白偶联受体30(Gprotein-coupled receptor, GPR30)表达增多所致，后者可以激活类固醇生成因子1(SF-1)，SF-1是一种可诱导子宫内膜细胞芳香化酶表达的转录因子。Robertson等[13]证实TAM可模拟雌激素作用与其他雌激素诱导的基因一起上调雌激素受体、c-fos、磷酸甘油醛脱氢酶mRNA的表达。TAM使用者子宫内膜细胞的溴脱氧尿苷(细胞有丝分裂指示物)含量是增加的，也可上调胰岛素样生长因子(IGF-1)和细胞增殖标志物如Ki67的表达水平[14]。另有研究认为TAM有增强黄体的功能，增加黄体期子宫内膜糖原合成以及雌激素和孕激素水平的表达，降低流产率[15]。在连续服用1个月TAM药物的女性，其子宫内膜上雌激素受体及孕激素受体表达水平更高[16]，有利于子宫内膜生长以及胚胎着床。17β-雌二醇与TAM诱导子宫内膜增生肥大的分子机理也存在差异。17β-雌二醇的大部分激动效应可被孕激素抵消；然而TAM的激动效应仅仅是部分与之抵消[17]。值得一提的是我们有13个周期患者在应用TAM过程中没有优势卵泡产生，但是子宫内膜仍然继续生长，可能与上述机理有关。这说明TAM有可能通过雌激素通路作用和非雌激素通路作用多途径来促使子宫内膜生长。

3.4 TAM应用的相关问题　在61例患者中，生化妊娠36例，其中临床妊娠27例，也就是有9例未能临床妊娠(25%,9/36)。通过查阅数据和病例我们发现在这9例患者中，有3例患者在移植时的内膜厚度是仍然小于7 mm的，虽然有好的胚胎，但是内膜因素仍然是制约临床妊娠的原因之一；有4例患者是PCO或者PCOS，2014年一篇文章认为多囊卵巢患者在行冻融胚胎移植时生化妊娠概率高于卵巢功能正常的患者，差异有统计学意义[18]。这可能是我们的生化妊娠率偏高的部分原因。本研究中出现3例异位妊娠，且患者现均存在异位妊娠的高危因素[19]。其中1例有输卵管积水病史，另外2例均有慢性盆腔炎、盆腔粘连病史。有研究发现，IVF后EP的发生率一般小于5%，但在输卵管因素不孕患者及曾有盆腔手术史中，EP发生率高达11%；由于样本量较少，TAM的使用是否会增加异位妊娠的机率有待进一步探讨。此外，我们研究发现TAM用于子宫内膜薄的患者行冻融胚胎移植是安全的，至今无一例发生出生缺陷，这主要是因为TAM的半衰期是86 h，而我们的TAM方案中，移植胚胎的时间至少在TAM停药后8 d。文献也报道妊娠期口服TAM后出生的婴儿是正常的[20]。

综上所述，对于既往自然周期和激素治疗周期出现子宫内膜薄的不孕患者，应用他莫昔芬准备子宫内膜进行冻融胚胎移植可以提高子宫内膜厚度、显著缩短子宫内膜准备时间，继而获得较为理想的临床妊娠率和胚胎着床率。本研究例数较少，有必要扩大临床样本进一步研究。基于对母体及子代其安全性考虑，是否可作为冻融胚胎移植的时子宫内膜准备的一线药物还有待进一步研究。

参 考 文 献

[1] Horne G, Critchlow JD, Newman MC, et al. A prospective evaluation of cryopreservation strategies in a two-embryo transfer programme[J]. Hum Reprod,1997,12(3):542-547.
[2] Chen S, Wu F, Luo C, et al. Combined analysis of endometrial thickness and pattern in predicting outcome of in vitro fertilization and embryo transfer: a retrospective cohort study[J]. Reprod Biol Endocrinol,2010, doi: 10.1186/1477-7827-8-30.
[3] Gargett CE, Healy DL. Generating receptive endometrium in Asherman´s syndrome[J]. J Hum Reprod Sci,2011,4(1):49-52.
[4] Gleicher N, Vidali A, Barad DH. Successful treatment of unresponsive thin endometrium[J]. Fertil Steril,2011,95(6):2113-2123.
[5] Wang H, Wang C, Horng S, et al. Ovulation induction with tamoxifen and alternate-day gonadotrophin in patients with thin endometrium[J]. Reprod Biomed Online,2008,17(1):20-26.
[6] Basir GS, O WS, So WW, et al. Evaluation of cycle-to-cycle variation of endometrial responsiveness using transvaginal sonography in women undergoing assisted reproduction[J]. Ultrasound Obstet Gynecol,2002,19(5):484-489.
[7] Senturk LM, Erel CT. Thin endometrium in assisted reproductive technology[J]. Curr Opin Obstet Gynecol,2008,20(3):221-228.
[8] Hutson PR. Effect of Exemestane on tamoxifen pharmacokinetics in postmenopausal women treated for breast cancer[J]. Clin Cancer Res,2005,11(24):8722-8727.
[9] Chen X, Chen S. Successful pregnancy in recurrent thin endome-

trium with new uses for an old drug[J]. J IVF Repord Med Genet,2013,1(2).Doi:10.4172/jfiv.1000110.
[10] Reynolds K, Khoury J, Sosnowski J, et al. Comparison of the effect of tamoxifen on endometrial thickness in women with thin endometrium (<7mm) undergoing ovulation induction with clomiphene citrate[J]. Fertil Steril,2010,93(6):2091-2093.
[11] Badawy A, Gibreal A. Clomiphene citrate versus tamoxifen for ovulation induction in women with PCOS: a prospective randomized trial[J]. Eur J Obstet Gynecol Reprod Biol,2011,159(1):151-154.
[12] Williams-Brown MY, Salih SM, Xu X, et al. The effect of tamoxifen and raloxifene on estrogen metabolism and endometrial cancer risk[J]. J Steroid Biochem Mol Biol,2011,126(3-5):78-86.
[13] Robertson JA, Bhattacharyya S, Ing NH. Tamoxifen up-regulates oestrogen receptor-alpha, c-fos and glyceraldehyde 3-phosphate-dehydrogenase mRNAs in ovine endometrium[J]. J Steroid Biochem Mol Biol,1998,67(4):285-292.
[14] Elkas J, Gray K, Howard L, et al. The effects of tamoxifen on endometrial insulin-like growth factor-1 expression[J]. Obstet Gynecol,1998,91(1):45-50.
[15] Wu CH. Less miscarriage in pregnancy following tamoxifen treatment of infertile patients with luteal phase dysfunction as compared to clomiphene treatment[J]. Early Pregnancy,1997,3(4):301-305.
[16] Cano A. Modulation of the oestrogen receptor: a process with distinct susceptible steps[J]. Hum Reprod Update,2000,6(3):207-211.
[17] Powles TJ, Bourne T, Athanasiou S, et al. The effects of norethisterone on endometrial abnormalities identified by transvaginal ultrasound screening of healthy post-menopausal women on tamoxifen or placebo[J]. Br J Cancer,1998,78(2):272-275.
[18] Liu L, Tong X, Jiang L, et al. A comparison of the miscarriage rate between women with and without polycystic ovarian syndrome undergoing IVF treatment[J]. Eur J Obstet Gynecol Reprod Biol,2014,176:178-182.
[19] Rana P, Kazmi I, Singh R, et al. Ectopic pregnancy: a review[J]. Arch Gynecol Obstet,2013,288(4):747-757.
[20] Berger JC, Clericuzio CL. Pierre Robin sequence associated with first trimester fetal tamoxifen exposure[J]. Am J Med Genet A,2008,146A(16):2141-2144.

（2015-02-10收稿　2015-05-04修回）

读者·作者·编者

《中国实用妇科与产科杂志》2015年第9期中心内容预告

中心内容：产前诊断技术与规范

- 遵循“规范”踏实前进 不断提高我国产前诊断水平
- 母体外周血胎儿游离核酸技术引入产前筛查策略——价值医学层面的思考
- 产时胎儿手术现状与展望
- 早中孕期血清学筛查方案适应证及应用
- 高通量测序技术在无创产前检测中的应用
- 细胞遗传学产前诊断技术与规范
- 植入前胚胎遗传学诊断及筛查技术与规范
- 产前超声检查及诊断规范化应用
- 胎儿磁共振在产前诊断中的应用及适应证
- 介入性产前诊断适应证 禁忌证及应用
- 双胎妊娠产前筛查和产前诊断
- TORCH以及人乳头瘤感染检测在孕前及孕期中的应用再评价
- 复发性流产相关染色体异常问题
