# Supplementary material for: Effect of tamoxifen in patients with thin endometrium who underwent frozen–thawed embryo transfer cycles: a retrospective study
Source: Front Endocrinol (Lausanne). 2023 Sep 1;14:1195181. doi: 10.3389/fendo.2023.1195181 (PMC10505727; doi:10.3389/fendo.2023.1195181)
Supplement: Supplementary file 2 [file DataSheet_2.pdf]

文章编号: 1003-6946(2016)10-0770-04

薄型子宫内膜患者冻融胚胎移植周期使用他莫昔芬治疗结局研究

刘景瑜,孔　娜,陈　华,王　玢

(南京大学医学院附属鼓楼医院,江苏 南京 210008)

【摘要】 目的: 探讨薄型子宫内膜患者冻融胚胎移植周期应用他莫昔芬(TAM)促排卵准备内膜的临床效果。方法: 回顾性分析 2014 年 1 月至 2015 年 10 月于我院进行冻融胚胎移植的薄型子宫内膜患者 205 例,其中 TAM 促排卵准备内膜组(TAM 组)113 例,激素替代组(HRT 组)92 例。比较两组间冻融胚胎(分裂胚及囊胚)移植周期内膜厚度、临床妊娠率、种植率、流产率。结果: TAM 组内膜明显厚于 HRT 组。TAM 组分裂胚的临床妊娠率、种植率明显高于 HRT 组($P<0.05$, $P<0.01$),流产率两组比较差异无统计学意义($P>0.05$),两组均无异位妊娠。两组间囊胚移植的临床妊娠率、种植率、流产率及异位妊娠率差异均无统计学意义($P>0.05$)。结论: 薄型子宫内膜患者冻融胚胎移植,推荐使用 TAM 促排卵及 HCG 诱导排卵方案准备内膜; 薄型内膜患者推荐囊胚优先移植。

【关键词】 冻融胚胎移植; 薄型子宫内膜; 他莫昔芬

中图分类号: R321-33　　文献标志码: A

Tamoxifen in Treatment of Thin Endometrium for Frozen-thawed Embryo Transfer Cycles

LIU Jingyu, KONG Na, CHEN Hua, et al

(Drum-tower Hospital Affiliated to Nanjing University, Nanjing Jiangsu 210008, China)

Corresponding author: WANG Bin

【Abstract】 Objective: To explore the effect of tamoxifen on the patients with thin endometrium in the frozen-thawed embryo transfer cycles. Methods: 205 patients with thin endometrium in the frozen-thawed embryo transfer cycles from January 2014 to October 2015 were retrospectively analyzed. 113 patients were given tamox-

通讯作者: 王　玢, Email: wang_bin429@sina.com

[2] Williams CJ. Signaling mechanisms of mammalian oocyte activation [J]. Hum Reprod Update, 2002, 8(4): 313-321.

[3] 徐　芬,徐望明,谢青贞. 体外受精后异常受精的影响因素分析 [J]. 中国优生与遗传杂志, 2009, 17(8): 114-116.

[4] Payne D, Flaherty S, Swann N, et al. Preliminary observations on polar body extrusion and pronuclear formation in human oocytes using time-lapse video cinematography [J]. Hum Reprod, 1997, 12(3): 532-541.

[5] Park EA, Park KH, Chung MK, et al. The developmental potential of human single pronuclear(1 PN) zygotes obtained from ICSI and conventional IVF [J]. Fertil Steril, 2002, 78(3): 237-238.

[6] Azevedo AR, Pinho MJ, Silva J, et al. Molecular cytogenetics of human single pronmcleated zygotes [J]. Reprod Sci, 2014, 21(12): 1472-1482.

[7] Porter R, Han T, Tucker MJ, et al. Estimation of second polar body retention rate after conventional insemination and intracytoplasmicsperm injection: in vitro observations from more than 5000 human oocytes [J]. Assist Reprod Genet, 2003, 20(9): 371-376.

[8] Findikli N, Kahraman S, SaglamY, et al. Embryo aneuploidy screening for repeated implantation failure and unexplained recurrent miscarriage [J]. Reprod Biomed Online, 2006, 13(1): 38-46.

[9] Gras L, Trounson AO. Pregnancy and birth resulting from transfer of ablastocyst observed to have one pronucleus at the timeof examination for fertilization [J]. Hum Reprod, 1999, 14(7): 1869-1871.

[10] Malcov M, Frumkin T, Shwartz T, et al. Elucidation of abnormal fertilization by single-cell analysis with fluorescence in situ hybridizationand polymorphic marker analysis [J]. Fertil Steril, 2009, 91(3): 932.

[11] 张亚楠,殷宝莉,张翠莲,等. 体外受精中 1 PN 及 0 PN(2 PB)胚胎发育潜能及影响因素分析 [J]. 生殖医学杂志, 2016, 25(1): 32-38.

[12] Manor D, Kol S, Lewit N, et al. Undocumented embryos: Do not trsh them, fish them [J]. Hum Reprod, 1996, 11(11): 2502-2506.

[13] 李　明,李军生,赵　平,等. 101 例 IVF-ET 新鲜周期移植未见原核来源胚胎临床结局分析 [J]. 中华妇产科临床杂志, 2013, 14(5): 427-429.

[14] 章师平,周　平,章志国,等. 未见原核的卵母细胞发育形成的优质胚胎的移植价值分析 [J]. 安徽医科大学学报, 2013, 48(1): 57-59.

(收稿日期: 2016-05-25; 修回日期: 2016-07-30)

(C)1994-2019 China Academic Journal Electronic Publishing House. All rights reserved.　http://www.cnki.net

ifen to prepare the endometrium (TAM group); 92 patients were given hormone replacement therapy (HRT group). The endometrial thickness, clinical pregnancy rate, implantation rate, and abortion rate were compared between the two groups. **Results:** The endometrium in TAM group were significantly thicker than that of HRT group. The cleavage embryo clinical pregnancy rates and implantation rates of TAM group were significantly higher than those of HRT group ($P<0.05$, $P<0.01$), while there was no significantly difference on the blastcyst clinical pregnancy rates, implantation rates, abortion rates, and ectopic pregnancy rates between the two groups ($P>0.05$). **Conclusions:** TAM and HCG were recommended for the ovulation induction in patients with thin endometrium in FET cycles. The blastcyst transfer was recommended for thin endometrial patients.

【Key words】 Frozen-thawed embryo transfer; Thin endometrium; Tamoxifen

自 1983 年首例冻融胚胎新生儿的出生[1]，冻融胚胎移植已成为辅助生殖技术必不可少的一部分。冻融胚胎移植周期内膜厚度对胚胎种植至关重要，而薄型子宫内膜的发生率约为 1%。研究发现他莫昔芬（tamoxifen，TAM）替代氯米芬（clomiphene，CC）用于促排卵治疗有促进内膜生长的作用[2]，将其应用于冻融胚胎移植周期的研究较少。本文探讨了薄型子宫内膜患者冻融胚胎移植周期应用 TAM 促排卵准备内膜的临床效果。

1 资料与方法

1.1 研究对象 回顾性分析 2014 年 1 月至 2015 年 10 月于我中心拟行冻融胚胎移植的薄型子宫内膜患者 205 例，分为两组。TAM 组：113 例患者既往超促排卵周期绒促性素（HCG）扳机日内膜厚度<8 mm，且至少 2 次激素替代周期因内膜<8 mm放弃移植，后改行 TAM 促排卵准备内膜；激素替代组（hormone replacement therapy，HRT 组）：92 例患者，超促排卵周期及 2 次激素替代周期内膜均<8 mm，但仍要求移植。每位患者仅纳入 1 个周期，且均移植优质冻融分裂胚或囊胚。不孕因素主要是输卵管因素，和（或）男方因素。子宫内膜异位症/子宫内膜腺肌病、子宫肌瘤、宫腔粘连或瘢痕均不纳入研究范围。所有患者均签署知情同意书，且经过本院医学伦理委员会同意认可。

两组患者年龄、不孕年限、不孕因素分布组间比较差异均无统计学意义（$P>0.05$）。见表 1。

表 1 HRT 组与 TAM 组临床资料比较

Tab 1 Comparison of clinical characteristics between HRT group and TAM group

	HRT 组（$n=92$）	TAM 组（$n=113$）	P
年龄（岁）	33.8±5.2	33.4±5.7	>0.05
不孕年限（y）	4.3±3.1	4.1±3.0	>0.05
不孕因素%（n）			
输卵管因素	59.8（55/92）	57.5（65/113）	>0.05
男方因素	23.9（22/92）	23.0（26/113）	>0.05
双方因素	16.3（15/92）	19.5（22/113）	>0.05

1.2 HRT 组内膜准备方案 月经周期第 3 天开始服用戊酸雌二醇（德国拜耳公司，补佳乐 1 mg/片）早晚各 3 mg，服用第 12～14 天，B 超监测内膜，若厚度≤7 mm，补佳乐加至 4 mg，每天 2 次，最多服用时间为 20 天，若内膜厚度<8 mm 可以要求放弃本周期。若要求移植，同时给予黄体酮 60 mg/d，5 天或 6 天后行解冻分裂胚或囊胚移植。移植后给予戊酸雌二醇早晚各 3 片，黄体酮 60 mg/d，至移植后 14 天测尿 HCG。

1.3 TAM 组内膜准备方案 月经周期第 3 天开始服用枸橼酸 TAM（扬子江药业，10 mg/片）20 mg/d，连续 5 天，月经周期第 9 天开始监测卵泡生长情况，当卵泡直径≥18 mm 时给予 HCG 10000 U 诱导排卵，诱导后第 5～7 天行解冻分裂胚或囊胚移植。诱导后第 2 天开始口服地屈孕酮（雅培，10 mg/片）20 mg，每天 2 次，同时给予 HCG 2000 U，每 3 天 1 次，共 2 次。若无卵泡发育则放弃本周期。

1.4 优质胚胎标准 分裂胚解冻后为Ⅰ级胚胎（细胞大小均等、透亮，胞质无颗粒，碎片≤5%）和Ⅱ级胚胎（细胞大小略不均，胞质可有颗粒现象，碎片 6%～20%）。根据 Garnder 囊胚分级法[3]解冻后 D5 评分≥3AA、3AB、3BA、3BB 或 D6～7 评分≥4AA、4AB、4BA、4BB 为优质囊胚。

1.5 妊娠结局评估 移植后 14 天验尿 HCG 阳性，移植后 30 天经阴道 B 超检查见妊娠囊为临床妊娠。

1.6 统计学分析 使用 SPSS 16.0 软件进行统计分析，均数采用均数±标准差（$\bar{x}\pm s$）表示，计数资料采用 Pearson χ^2 检验，计量资料采用 t 检验，以 $P<0.05$ 为差异有统计学意义。

2 结　果

HRT 组移植胚胎数 1.7±0.5 个，TAM 组移植胚胎数 1.7±0.4 个，两组比较差异无统计学意义（$P>0.05$）。HRT 组分裂胚移植周期 52 个，囊胚移植周期 40 个；TAM 组分裂胚移植周期 75 个，囊胚移植周期 38 个；将分裂胚及囊胚周期分开比较。无论移植分裂胚或囊胚，两组移植前内膜厚度差异均有统计学意义（$P<0.01$），TAM 组明显较厚，且较其既往放弃 HRT 周期明显增厚。TAM 组分裂胚移植的临床妊娠率、种

(C)1994-2019 China Academic Journal Electronic Publishing House. All rights reserved. http://www.cnki.net

植率明显高于 HRT 组（$P<0.05$，$P<0.01$），流产率两组比较差异无统计学意义（$P>0.05$），两组均无异位妊娠。两组间囊胚移植的临床妊娠率、种植率、流产率及异位妊娠率差异均无统计学意义（$P>0.05$）。见表 2、表 3。

表 2　HRT 组与 TAM 组冻融分裂胚临床结局比较

Tab 2　Comparison of clinical outcomes between HRT group and TAM group in frozen-thawed embryo transfer cycles

	HRT 组	TAM 组	TAM 组既往 HRT 放弃周期	P
周期数	52	75	–	–
移植前内膜厚度（mm）	7.1±0.3	8.2±1.1	6.6±0.7	<0.01①
临床妊娠率%（n）	38.5（20/52）	48.0（36/75）	–	<0.05
胚胎种植率%（n）	28.7（27/94）	34.6（47/136）	–	<0.01
流产率%（n）	20.0（4/20）	16.7（6/36）	–	>0.05②

①HRT 组与 TAM 组间比较，TAM 组和其既往 HRT 放弃周期间比较：P 均<0.01；②卡方检验连续性校正

表 3　HRT 组与 TAM 组冻融囊胚临床结局比较

Tab 3　Comparison of clinical outcomes between HRT group and TAM group in frozen-thawed blastocyst transfer cycles

	HRT 周期移植组	TAM 组	TAM 组既往 HRT 周期	P
周期数	40	38	–	–
移植前内膜厚度（mm）	6.9±0.2	8.3±1.2	6.5±0.7	<0.01①
临床妊娠率%（n）	45.0（18/40）	47.4（18/38）	–	>0.05
胚胎种植率%（n）	40.0（24/60）	43.3（26/60）	–	>0.05
流产率%（n）	5.6（1/18）	0（0/18）	–	>0.05②
异位妊娠率%（n）	5.6（1/18）	5.6（1/18）	–	>0.05②

①HRT 组与 TAM 组间，TAM 组和其既往 HRT 放弃周期间 P 均<0.01；②卡方检验连续性校正

3　讨　论

我国不孕症发病率约为 10%，且有逐年上升趋势。目前辅助生殖技术是解决不孕症最主要的手段，其中体外受精-胚胎移植（IVF-ET）技术已被广泛应用。IVF-ET 术后多余胚胎的冻融移植技术（FET）是辅助生殖技术必不可少的一部分，是增加胚胎利用率，提高累积妊娠率，减轻患者负担的有效方法。冻融胚胎移植周期内膜准备很重要，足够的内膜厚度对胚胎种植很关键[4]。目前对于薄型内膜的定义无统一标准，内膜在 7～8 mm 以下通常被认为不适宜胚胎移植，会降低妊娠率[5]。结合我中心 B 超测量内膜的标准，本文将超促排卵周期 HCG 日及 2 次激素替代周期内膜<8 mm，且排除宫腔粘连或瘢痕定为薄型内膜标准。约 1% 的薄型内膜患者表现为慢性内膜抵抗，即对任何治疗方法均无反应，在临床上较为棘手，严重影响 FET 的成功率，给患者带来心理及经济负担。

TAM 与 CC 类似，是一种人工合成的可与雌激素受体结合的非激素类制剂，是一种选择性雌激素受体调节剂，对不同靶器官具有雌激素和（或）抗雌激素样双重作用[6]，临床上主要用于乳腺癌术后辅助治疗。TAM 与雌激素受体结合后反馈性促使下丘脑和垂体分泌促性激素释放激素和促性腺激素，起到促排卵作用。1971 年，Klopper 与 Hall 首次将 TAM 应用于促排卵治疗。此后的多项研究发现，TAM 与 CC 的促排卵机制及效果相似，但 TAM 对子宫内膜具有类雌激素效应，能促使子宫内膜生长，使用 TAM 周期的内膜厚度均明显厚于 CC 周期[7～10]。辅助生殖技术方面，有学者在人工授精（IUI）周期使用 TAM 联合尿促性素（HMG）促排卵发现，相对于 CC 联合 HMG，TAM 组的内膜厚度明显增加，流产率明显降低[11]。

基于以上研究的提示，我们将 TAM 用于薄型子宫内膜冻融胚胎移植周期的内膜准备。回顾性分析我中心 2014 年 1 月至 2015 年 10 月的 205 例薄型子宫内膜患者 FET 周期。其中 92 例在 HRT 周期内膜<8 mm 的情况下坚持要求移植，113 例患者选择放弃 HRT 周期，改行 TAM。结果发现改用 TAM 后的内膜较 HRT 周期明显增厚（$P<0.05$），与既往研究结果一致[11]。冻融分裂胚胎移植的临床妊娠率及种植率 TAM 组明显高于 HRT 组，差异有统计学意义；囊胚种植率 TAM 组略高于 HRT 组，但差异无统计学意义。数据显示两组囊胚种植率都高于分裂胚（HRT 组：40% vs 28.7%；TAM 组：43.3% vs 34.6%），流产率均低于分裂胚（HRT 组：5.6% vs 20.0%，TAM 组：0 vs 16.7%），可见囊胚种植能力较强。TAM 组囊胚移植结局并未显示出优势，一方面可能是由于样本量较小，另一方面推测囊胚较强的种植能力弥补了薄型内膜对胚胎种植的不利影响，因此对于既有分裂胚又有囊胚冷冻的薄型子宫内膜患者推荐优先移植冻融囊胚。

结合既往文献及我中心数据认为 TAM 能明显改善薄型子宫内膜的厚度，增加分裂胚胎种植率。其中的机制可能是多方面的。首先，内膜的生长需要适量的子宫血流，研究表明薄型内膜的病理生理学特点可能由于子宫内膜血流灌注减少影响腺上皮的生长，导致血管内皮生长因子（VEGF）表达的减少，VEGF 是内膜血管生长调节的关键因子，结果导致内膜血管的损伤，进一步降低血流灌注，形成恶性循环，导致薄型内膜[12]。而研究发现 TAM 可以上调子宫内膜 VEGF 的表达，促进子宫内膜微血管的形成[13]。

改善内膜微血管的同时，TAM 促排卵周期优势卵

(C)1994-2019 China Academic Journal Electronic Publishing House. All rights reserved.　http://www.cnki.net

泡发育，诱导排卵后黄体形成，而 TAM 有增强黄体功能，增加黄体期子宫内膜糖原合成以及雌孕激素水平表达，降低流产率的作用[14,15]。此外，使用 HCG 诱导优势卵泡排卵，也对胚胎种植有一定的帮助。HCG 是调控胚胎种植的重要因子之一。它启动并控制着种植期绒毛膜胎盘的建立，同时调节母体对胚胎的免疫耐受[16,17]。分泌期子宫内膜上表达 HCG 受体，使用 HCG 能上调白细胞抑制因子(LIF)、VEGF 和基质金属蛋白酶-9(MMP-9)等的表达[18,19]，以上因子均能增加子宫内膜的容受性，改善内膜功能。

综上所述，对于顽固的薄型子宫内膜患者冻融胚胎移植，推荐可尝试 TAM 促排卵及 HCG 诱导排卵方案准备内膜，能改善内膜厚度，增加分裂胚胎种植率，降低流产率。薄型内膜患者推荐囊胚优先移植。作为促排卵用药 TAM 也是安全的，研究显示短时间应用 TAM 不会增加胎儿畸形风险，也不会增加卵巢癌或子宫内膜癌的风险[20]。但由于本研究样本量较小，能否将 TAM 作为冻融胚胎移植的一线用药仍需要大样本量的研究。

参 考 文 献

[1] Trounson A, Mohr L. Human pregnancy following cryopreservation, thawing and transfer of an eight cell embryo [J]. Nature, 1983, 305(5936): 707-709.

[2] Steiner AZ, Terplan M, Paulson RJ. Comparison of tamoxifen and clomiphene citrate for ovulation induction: a meta-analysis [J]. Hum Reprod, 2005, 20(6): 1511.

[3] Gardner DK, Lane M, Stevens J, et al, Blastocyst score affects implantation and pregnancy outcome: towards a single blastocyst transfer [J]. Fertil Steril, 2000, 73(6): 1155-1158.

[4] Mc Williams GD, Frattarelli JL. Changes in measured endometrial thickness predict in vitro fertilization success [J]. Fertil Steri, 2007, 88(1): 74-81.

[5] Zhang X, Chen CH, Confino E, et al. Increased endometrial thickness is associated with improved treatment outcome for selected patients undergoing in-vitro fertilization-embryo transfer [J]. Fertil Steri, 2005, 83(1): 336-340.

[6] Oehler MK, Rees MC, Bicknel R. Steroids and the endometrium [J]. Curr Med Chem, 2000, 7(5): 543-560.

[7] Reynolds K, Khoury J, Sosnowski J, et al. Comparison of the effect of tamoxifen on endometrial thickness in women with thin endometrium (<7mm) undergoing ovulation induction with clomiphen ecitrate [J]. Fertil Steril, 2010, 93(3): 2091-2093.

[8] Dhallwal LK, Suri V, Gupta KR, et al. Tamoxifen: an alternative to clomiphene in women with polycystic ovary syndrome [J]. J Hum Reprod Sci, 2011, 4(2): 76-79.

[9] Seyedoshohadaei F, Zandvakily F, Shahgeibi S. Comparison of the effectiveness of clomiphene citrate, tamoxifen and letrozole in ovulation induction in infertility due to isolated unovulation [J]. Iran J Reprod Med, 2012, 10(6): 531-536.

[10] El-Gharib MN, Mahfouz AE, Farahat MA. Comparison of letrozole versus tamoxifen effects in clomiphen citrate resistant women with polycystic ovarian syndrome [J]. Reprod Infertil, 2015, 16(1): 30-35.

[11] Wang CW, Horng SG, Chen CK, et al. Ovulation induction with tamoxifen and alternate-day gonadotrophin in patients with thin endometrium [J]. Reproductive Bio Medicine, 2008, 17(1): 20-26.

[12] Miwa I, Tamura H, Takasaki A, et al. Pathophysiologic features of "thin" endometrium [J]. Fertil Steril, 2009(91): 998-1004.

[13] 杨保军，邢凤玲，冯力民，等. TAM 对子宫内膜血管内皮生长因子表达的影响 [J]. 首都医科大学学报, 2007, 28(3), 412-414.

[14] Wu CH. Less miscarriage in pregnancy following tamoxifen treatment of infertile patients with luteal phase dysfunction as compared to clomiphene treatment [J]. Early Pregnancy, 1997, 3(4): 301-305.

[15] Fukushima T, Tajima C, Fukuma K, et al. Tamoxifen in treatment of infertility associated with luteal phase [J]. Fertil Steril, 1982, 37(6): 755-761.

[16] Tsampalas M, Gridelet V, Berndt S, et al. Human chorionic gonadotropin: a hormone with immunological and angiogenic properties [J]. J Reprod Immunol, 2010, 85(1): 93-98.

[17] Zenclussen AC, Gerlof K, Zenclussen ML, et al. Regulatory T cells induce a privileged tolerant microenvironment at the fetal-maternal interface [J]. Eur J Immunol, 2006, 36(1): 82-94.

[18] Fogle RH, Li A, Paulson RJ. Modulation of HOXA10 and other markers of endometrial receptivity by age and human chorionic gonadotropin in an endometrial explant model [J]. Fertil Steril, 2010, 93(4): 1255-1259.

[19] Licht P, Fluhr H, Neuwinger J, et al. Is human chorionic gonadotropin directly involved in the regulation of human implantation [J]. Mol Cell Endocrinol, 2007, 269(1-2): 85-92.

[20] Berger JC, Clericuzio CL. Pierre robin sequence associated with first trimester fetal tamoxifen exposure [J]. Am J Med Gen-et A, 2008, 146(16): 2141-2144.

(收稿日期: 2016-06-22; 修回日期: 2016-08-03)

启 事

请投稿作者投稿时务必登录本刊网址: JPOG. scyx. org. cn 注册成功后投稿。投稿作者注册投稿后可打电话(028-86131263)至本刊编辑部查证稿件是否被本刊收到。注册时，本刊仅收 50 元稿件审理费，并要求通过邮局邮政汇款寄至本刊编辑部收。本刊收稿需 1 个月后才能初步确定稿件是否被专家审查通过。此外，稿件还需经编辑部加工、返回作者本人修改等多项工序才能达到出版要求。稿件可以刊用后，本刊方通知投稿作者办理交费事宜，所有费用均经过邮局邮政汇款(汇款地址: 成都市武侯区玉林南街 2 号附 3 号; 收款人:《实用妇产科杂志》编辑部; 邮编: 610041)。本刊从不接受任何银行电子汇款。

(C)1994-2019 China Academic Journal Electronic Publishing House. All rights reserved. http://www.cnki.net
